# Does lack of exposure to individual antidepressants at different points during pregnancy associate with reduced risk of adverse newborn outcomes?

**DOI:** 10.1186/s12884-022-05287-6

**Published:** 2022-12-09

**Authors:** Margaret A. Tharp, Rebecca M. Silvola, Claire Marks, Evgennia Teal, Sara K. Quinney, David M. Haas

**Affiliations:** 1 Department of Obstetrics and Gynecology, Indiana University School of Medicine, 550 N. University Blvd, UH 2440, Indianapolis, IN 46033 USA; 2 Department of Medicine, Division of Clinical Pharmacology, Indiana University School of Medicine, Indianapolis, IN USA; 3 Regenstrief Institute, Indianapolis, IN USA

**Keywords:** Antidepressants, Pregnancy, Adaptation syndrome, Newborn intensive care unit admission, Trimester

## Abstract

**Background:**

The objective of this study was to determine if the lack of exposure to individual antidepressants at certain times in pregnancy improved maternal and infant outcomes.

**Methods:**

This was a retrospective cohort study of 2741 pregnant women prescribed antidepressant(s) before or during pregnancy. Data were obtained from electronic medical records. Analysis was limited to women prescribed one of five antidepressants (bupropion, citalopram, escitalopram, fluoxetine, sertraline). Period of exposure was determined using prescription order dates. Primary outcomes were neonatal intensive care unit (NICU) admission and adaptation syndrome in the newborn. Logistic regression, adjusted for maternal age, race, and insurance, compared consistent exposure throughout pregnancy versus (A) no exposure in the third trimester, (B) no exposure early in pregnancy, and (C) exposure in the midtrimester alone.

**Results:**

Compared to women prescribed an antidepressant continually throughout pregnancy, NICU admission was less likely for women lacking exposure in the third trimester if they had been taking bupropion (aOR 0.43, 95% CI 0.21–0.90) or escitalopram (aOR 0.49, 95% CI 0.28–0.85). Women previously taking escitalopram but lacking third trimester exposure also had lower odds of adaptation syndrome (aOR 0.19, 95% CI 0.07–0.48). No differences were found in other outcomes for women taking other antidepressants or for any outcomes for women who lacked early pregnancy drug exposure compared to exposure throughout pregnancy.

**Conclusion:**

For the five antidepressants included in this study, lack of exposure early or late in pregnancy compared to consistent exposure throughout pregnancy generally did not change newborn outcomes. The exceptions were bupropion and escitalopram, where lack of exposure in the third trimester associated with lower rates of adaptation syndrome or NICU admission. These data may help pregnant women with depression in need of drug therapy to have informed discussions with providers about the potential risks and benefits to continuing or stopping drugs at different times during pregnancy.

**Supplementary Information:**

The online version contains supplementary material available at 10.1186/s12884-022-05287-6.

## Introduction

Affecting more than 13% of pregnant women, maternal depression is common and can present significant and potentially debilitating challenges to mothers and infants both during and after pregnancy [1]. Untreated depression during pregnancy is associated with adverse outcomes, including increased risk of preterm delivery, preeclampsia, low birth weight, infant behavior disturbances at birth, fetal growth restriction, and maternal suicide [2–4]. Antidepressant medications, including selective serotonin reuptake inhibitors and selective norepinephrine reuptake inhibitors (SSRIs and SNRIs, respectively), can be effective for mothers experiencing the challenges of depression during pregnancy. Up to 71% of pregnant patients with depression may take medications as part of therapy [5].

Studies assessing the impact of exposure to antidepressants as a drug class on pregnancy outcomes have been conducted, however the typical exclusion of pregnant women from clinical trials during the drug development process results in relatively little research conducted on specific antidepressant medications in this population. While prior work has touched on variability in rates of some newborn outcomes associated with different SSRI/SNRI drugs used in different trimesters, gaps in granular data for individual drugs and exposure timing remain [6, 7]. Though potentially vital to the mental health of pregnant women with depression, some studies have found antidepressant exposure during pregnancy to be associated with preterm birth, low birth weight, adaptation syndrome, pulmonary complications, and cardiac malformations in the infant [6]. The tradeoff between risk of untreated depression and exposure to antidepressant medications at certain times throughout pregnancy remains unclear. These considerations present a potential area of further study to look more closely at how the timing of exposure during pregnancy to a specific antidepressant might be related to certain adverse outcomes. As opinions about drug therapy during the first and third trimesters of pregnancy continue to conflict, clinicians and patients would benefit from additional data to help guide clinical treatment decisions relating to these drugs.

Thus, the objective of this study was to compare associations between timing of potential exposure to individual specific SSRI or SNRI antidepressants and maternal and infant outcomes. Our hypothesis was that not having exposure (potentially meaning starting or stopping) an antidepressant later in pregnancy, compared to continued use throughout pregnancy, would have significantly different maternal and newborn outcomes. Findings such as this could be clinically useful when counseling pregnant women with depression who may benefit from SSRI or SNRI treatment.

## Methods

This was a retrospective cohort study of women prescribed antidepressants before and during pregnancy. This study was approved by the Indiana University Institutional Review Board and Regenstrief Institute in Indianapolis. Deidentified data were obtained from electronic medical records (EMRs) through the Regenstrief Institute in Indianapolis, an honest data broker for EMR data from multiple large health systems in Indiana [8, 9]. Data related to pregnancies within Eskenazi Health or Indiana University Health Systems between January 1, 2010, and December 31, 2019 were collected. Medication orders (i.e. prescriptions) for SSRI or SNRI dated 100 days before the last menstrual period through the date of delivery were captured. Antidepressants of interest included the SSRIs citalopram, escitalopram, fluoxetine, and sertraline as well as the SNRI bupropion. Data on the SSRI paroxetine and SNRIs desvenlafaxine, duloxetine, and venlafaxine were collected and ultimately excluded from analyses due to the number of subjects with orders for these medications at timepoints of interest failing to reach *n* = 200, limiting statistical ability to effectively detect rates for the outcomes of interest. Tricyclic and tetracyclic antidepressants were also excluded due to being less commonly prescribed during pregnancy for our population. This was a planned secondary analysis focusing on timing of prescriptions of a larger antidepressant database [7].

The following variables were collected for eligible women: maternal age at time of delivery, race, ethnicity, insurance, estimated due date, history of prior preterm birth, any other drugs prescribed during pregnancy, history of diabetes (types I, II), development of gestational diabetes (GDM), and development of a hypertensive disorder of pregnancy. Infant outcomes collected were gestational age at birth, birth weight, birth length, birth head circumference, stillbirth, diagnosis of any adaptation syndrome, neonatal intensive care unit (NICU) admission, 5-minute APGAR score, jaundice requiring treatment, diagnosis of transient tachypnea of the newborn (TTN) or respiratory distress syndrome (RDS), persistent pulmonary hypertension of the newborn (PPHN), neonatal seizures, and cardiac malformations.

The primary outcomes of interest were newborn diagnosis of any adaptation syndrome and NICU admission. Due to a change in hospital coding for the diagnosis, coded diagnoses of “neonatal abstinence syndrome” (NAS) or “pediatric adaptation syndrome” (PAS) were combined for the diagnosis of any adaptation syndrome. Discharge summaries, delivery records, and administrative ICD codes were used to extract diagnoses. Appendix Table S1 lists the codes and sources used by the data extraction process. Extraction of clinical diagnoses was contingent upon use of standard clinical criteria documented by the clinician in the discharge summary of an infant. The Regenstrief Institute Data Core’s documented process based on ICD9/10 codes was utilized for capture of diagnoses [8, 9]. Through the linkage of maternal and infant medical record numbers across health systems, all diagnoses and diagnostic codes for the infant were extracted by the Data Core for both inpatient and outpatient encounters. Identification and verification for accuracy against the Cerner or Epic medical records of the respective health system was completed for approximately 1% of records [7]. Data for these subjects was then re-deidentified for the final analyses.

The data were aggregated and organized using SPSS 26 and Microsoft Excel and analyzed using SPSS 28. Data were analyzed using chi-square testing for discreet variables. Logistic regression models, adjusting for maternal age, race, ethnicity, and insurance status were used to determine the impact of prescription timing on the primary outcomes.

Because the objective of this study was to screen for any impact on maternal and infant outcomes based on timing of antidepressant exposure, women who were prescribed an antidepressant within 100 days before the last menstrual period, as well as in first (0–14 weeks gestation), second (15–28 weeks), and third trimesters (> 28 weeks) of their pregnancy were used as the control group (i.e. subjects with assumed continual medication exposure throughout pregnancy). Exposure timing was extrapolated based on the date the prescription was ordered. As we did not have data on whether the person actually took the medication, we assumed that the drug was taken and there was exposure.

Three separate comparisons were performed to explore the impact of timing and lack of exposure to each of the drugs. Consistent exposure throughout pregnancy was compared to 1) exposure up to the third trimester (no prescriptions in the third trimester), 2) exposure starting in the second trimester (no record of prescriptions before pregnancy or during first trimester), and 3) exposure occurring only during the second trimester (prescriptions dated during second trimester alone; potentially representing women without exposure in the first and third trimesters). All three exposure timing groups were assessed for each drug. Women who delivered before the third trimester were excluded from comparison 1. Logistic regression models, adjusting for maternal age, race, and primary insurance payor, were then constructed to examine the impact of timing of drug exposure on the primary outcomes. These results were reported in adjusted odds ratios (aOR) and 95% confidence intervals (CI).

## Results

From the initial data set of 3694 women with a single pregnancy, 2741 women had prescription orders for at least one of the five drugs of interest (bupropion, citalopram, escitalopram, fluoxetine, sertraline) in the time surrounding a pregnancy (Table 1). The distribution of drugs taken included bupropion (*n* = 315, 11.5%), citalopram (*n* = 308, 11.2%), escitalopram (*n* = 470, 17.1%), fluoxetine (n = 470, 17.1%), and sertraline (*n* = 1443, 52.6%). There were 262 women with prescription orders for more than one of the drugs of interest, totaling to 3006 potential drug exposures. The majority (80.9%) of women in the study were White (*n* = 2219), 25.6% were Black (*n* = 701), 0.6% were Asian (*n* = 17), and 0.5% were categorized as other or did not identify their race (*n* = 15). Hispanic ethnicity was reported in 103 (3.8%) of women. Insurance was listed as government (*n* = 1285, 46.9%), commercial (*n* = 1490, 54.3%), and self-pay or unknown (*n* = 54, 2.0%) and some women had multiple forms of insurance recorded. Of the 2741 women in this secondary study, 133 had preexisting diabetes mellitus (4.8%), 169 (6.2%) had hypertension, and 112 had history of preterm birth (4.1%) at baseline. There were 274 women who developed gestational diabetes (10.0%) and 489 who developed hypertensive disorders of pregnancy (17.8%) (Table 1). Demographic characteristics and outcomes were tested for differences among the five drugs of interest and noted in Table 1. The age for women taking each drug ranged from 23.2 to 34.8 years and was significantly different between drugs (*p* < 0.001) (Table 1).

**Table 1:** Maternal characteristics and maternal/infant outcomes for women prescribed antidepressant(s) at any point throughout pregnancy

Cohort (***N*** = 2741)	Bupropion	Citalopram	Escitalopram	Fluoxetine	Sertraline	Overall cohort
**Drug Frequency (women with drug orders)**	315 (11.5%)	308 (11.2%)	470 (17.1%)	470 (17.1%)	1443 (52.6%)	3006
**Mean maternal age (sd)**	29.8 (5.9)	29.9 (5.7)	29.4 (5.5)	29.0 (6.0)	28.5 (5.8)	29.0 (5.8)
Exposure Profile*^,b^						
**Throughout pregnancy**	56 (17.8%)	58 (18.8%)	86 (18.3%)	90 (19.1%)	268 (18.6%)	558 (18.6%)
**Pre-pregnancy, 1st, & 2nd trimesters**	23 (7.3%)	21 (6.8%)	34 (7.2%)	23 (4.9%)	103 (7.1%)	204 (6.8%)
**Pre-pregnancy & 1st trimester**	29 (9.2%)	36 (11.7%)	52 (11.1%)	52 (11.1%)	114 (7.9%)	283 (9.4%)
**Pre-pregnancy & 2nd trimester**	15 (4.8%)	16 (5.2%)	22 (4.7%)	24 (5.1%)	62 (4.3%)	139 (4.6%)
**2nd & 3rd trimesters**	25 (7.9%)	23 (7.5%)	28 (6.0%)	37 (7.9%)	95 (6.6%)	208 (6.9%)
**Pre-pregnancy**	22 (7.0%)	29 (6.4%)	30 (3.4%)	30 (6.4%)	68 (4.7%)	179 (5.6%)
**2nd trimester**	54 (17.1%)	28 (10.4%)	49 (10.4%)	66 (14.0%)	214 (14.8%)	411 (13.7%)
**3rd trimester**	74 (23.5%)	67 (21.7%)	127 (27.0%)	107 (22.8%)	393 (27.2%)	768 (25.5%)
Race*						
**Asian**	1 (0.3%)	1 (0.3%)	1 (0.2%)	5 (1.1%)	9 (0.6%)	17 (0.6%)
**Black**	23 (7.3%)	27 (8.8%)	426 (90.6%)	54 (11.5%)	171 (11.9%)	701 (25.6%)
**White**	290 (92.1%)	274 (89.0%)	37 (7.9%)	395 (84.0%)	1223 (84.8%)	2219 (80.9%)
**Other/Not Identified**	1 (0.3%)	1 (0.3%)	1 (0.2%)	4 (0.9%)	8 (0.6%)	15 (0.5%)
**Ethnicity**						
**Non-Hispanic/Latina**	311 (98.7%	295 (95.8%)	459 (97.7%)	449 (95.5%)	1389 (96.3%)	2903 (96.2%)
**Hispanic/Latina**	4 (1.3%)	13 (4.2%)	11 (2.3%)	21 (4.5%)	54 (3.7%)	103 (3.8%)
Hospital*						
**Eskenazi**	15 (4.8%)	19 (6.2%)	17 (3.6%)	27 (5.7%)	116 (8.0%)	194 (6.4%)^a^
**IU Health**	300 (95.2%)	289 (93.8%)	453 (96.4%)	443 (94.3%)	1327 (92.0%)	2812 (93.5%)^a^
Insurance*						
**Commercial**	171 (54.3%)	140 (45.5%)	269 (57.2%)	232 (4.4%)	678 (47.0%)	1490 (54.3%)
**Government**	140 (44.4%)	165 (53.6%)	196 (41.7%)	226 (48.1%)	735 (50.9%)	1285 (46.9%)
**Self-Pay/None**	4 (1.3%)	3 (1.0%)	5 (1.1%)	12 (2.6%)	30 (2.1%)	54 (2.0%)
**Maternal Pregnancy Complications**						
**GDM**	35 (11.1%)	28 (9.1%)	36 (7.7%)	43 (9.1%)	132 (9.1%)	274 (10.0%)
**HDP**	66 (21.0%)	50 (16.2%)	69 (14.7%)	76 (16.2%)	228 (15.8%)	489 (17.8%)
**Infant Outcomes**						
Any Adaptation Syndrome*	16 (5.1%)	30 (9.7%)	47 (10.0%)	29 (6.2%)	71 (4.9%)	193 (7.0%)
NICU Admission*	74 (23.5%)	90 (29.2%)	147 (31.3%)	139 (29.6%)	345 (23.9%)	795 (29.0%)
**Preterm Birth**	50 (15.9%)	45 (14.6%)	72 (15.3%)	86 (8.3%)	203 (14.1%)	456 (16.6%)
TTN*	28 (8.9%)	41 (13.3%)	60 (12.8%)	75 (16.0%)	153 (10.6%)	357 (13.0%)

Data presented as n (% with characteristic or outcome) in individual groups. 2741 women were in the cohort, with 262 who were prescribed more than one drug, accounting for 3006 drug prescriptions total

Denominator used for % are for women in the cohort or for women exposed to each drug, unless denoted by ^a^ where the denominator for the overall cohort column is by the 3006 drug exposures

*Statistically significant rate differences of outcome distribution between the drugs (*p* < 0.05)

^b^Exposure profile based on when prescription orders were placed for the women relative to their estimated due date with pre-pregnancy being up to 100 days before the date of the last menstrual period, first trimester being the last period up to 14 weeks gestation, second trimester being 15–28 weeks’ gestation, and third trimester being after 28 weeks’ gestation. % given for number of women with exposure at the different times for that drug

*sd* standard deviation, *GDM* gestational diabetes, *HDP* hypertensive disorder of pregnancy, *NICU* neonatal intensive care unit, *TTN* transient tachypnea of the newborn

Table 1 also displays the outcome rates associated with individual drug exposure compared to the overall cohort. One hundred ninety-three women (7.0%) had neonates diagnosed with any adaptation syndrome, 795 (29.0%) had neonates admitted to the NICU, 456 (16.6%) had a preterm birth, and 357 (13.0%) had neonates diagnosed with TTN. The rates of these outcomes, except preterm birth, were different between the five drugs (*p* < 0.05). Other newborn characteristics were not different between drugs and are reported in Appendix Table S2.

There were 558 women noted to have prescriptions ordered throughout the entire pregnancy (18.6%, Table 2). For comparison 1, 1216 women (40.5%) had prescriptions that lacked orders dated in the third trimester, representing women who may not have had third trimester exposure. For comparison 2, 1387 women (46.1%) had no prescriptions for the individual medication ordered until the second trimester, representing women who did not have early pregnancy exposure. For comparison 3, 411 women (13.7%) had prescription orders dated within in the second trimester alone, representing women who may have been without exposure in the early and/or late pregnancy period. Excluding patients diagnosed with diabetes or a hypertensive disorder did not significantly change the results so Table 2 displays the regression for the entire cohort.

**Table 2:** Comparison of key newborn complication outcomes for the five selected antidepressants comparing exposure throughout pregnancy to no exposure in certain trimesters

Odds ratios for exposure timepoints – Adjusted for age, race, and insurance status					
Drug	Outcome	All timepoints	Comparison 1: No third trimester exposure	Comparison 2: No early exposure	Comparison 3: mid-pregnancy exposure only
**Bupropion**	**Adaptation syndrome**	*Reference*	1.24 (0.28–5.41)	1.31 (0.33–5.16)	1.78 (0.32–10.08)
	**NICU admission**	*Reference*	0.43 (0.21–0.91)^a^	0.75 (0.37–1.49)	0.49 (0.19–1.23)
**Citalopram**	**Adaptation syndrome**	*Reference*	0.79 (0.22–2.90)	2.54 (0.77–8.30)	0.87 (0.14–5.28)
	**NICU admission**	*Reference*	0.97 (0.44–2.14)	1.09 (0.52–2.32)	1.06 (0.33–3.37)
**Escitalopram**	**Adaptation syndrome**	*Reference*	0.19 (0.07–0.48)^a^	0.54 (0.25–1.18)	0.12 (0.02–0.97)
	**NICU admission**	*Reference*	0.49 (0.28–0.85)^a^	0.59 (0.35–1.02)	0.45 (0.20–1.01)
**Fluoxetine**	**Adaptation syndrome**	*Reference*	0.54 (0.16–1.87)	0.97 (0.35–2.69)	0.24 (0.03–2.10)
	**NICU admission**	*Reference*	1.11 (0.62–2.00)	1.57 (0.89–2.78)	1.62 (0.78–3.37)
**Sertraline**	**Adaptation syndrome**	*Reference*	0.53 (0.25–1.11)	0.92 (0.48–1.74)	0.45 (0.16–1.22)
	**NICU admission**	*Reference*	0.96 (0.69–1.37)	1.06 (0.75–1.49)	0.85 (0.54–1.34)

Adjusted logistic regression controlled for maternal age, race, and insurance status

Results reported as adjusted odds ratio (aOR) (95% confidence interval)

^a^Confidence interval does not cross 1.0, indicating a statistically significant result

All timepoints = pre-pregnancy, first, second, and third trimester prescriptions; Early pregnancy = pre-pregnancy, first and second trimester prescriptions; Late pregnancy = second and/or third trimester prescriptions; Mid-pregnancy = second trimester prescriptions; NICU = neonatal intensive care unit

Table 2 (Comparison 1) summarizes, after adjusting for maternal age, race, and insurance status, women with prescriptions ordered consistently throughout pregnancy compared to those who lacked prescriptions in the third trimester. Comparison 1 demonstrated that women lacking third trimester exposure were less likely to have neonates admitted to the NICU if taking bupropion (aOR 0.43, 95% CI 0.21–0.90) or escitalopram (aOR 0.49, 95% CI 0.28–0.85). Additionally, infants of mothers taking escitalopram but lacking third trimester exposure were less likely to develop adaptation syndrome (aOR 0.19, 95% CI 0.07–0.48).

Table 2 (Comparison 2) demonstrates that, compared to women who had prescriptions ordered continually throughout pregnancy, those who lacked prescription orders early in pregnancy did not have any significant associated differences in outcomes for any of the individual drugs after adjustment for maternal age, race, and insurance.

Table 2 (Comparison 3) illustrates that, compared to women who had prescriptions ordered continually throughout pregnancy, women prescribed escitalopram had lower odds of adaptation syndrome if they had only mid-trimester exposure (aOR 0.12, 95%CI 0.02–0.97). No other differences were noted for women taking any of the drugs for other outcomes if exposure occurred strictly in the mid-trimester alone.

## Discussion

This analysis of individual SSRI/SNRI drug exposures found that, compared to women prescribed antidepressants consistently throughout their pregnancy, women lacking bupropion or escitalopram prescriptions dated for the third trimester had associations with lower rates of infants requiring NICU admission. Additionally, women who were prescribed escitalopram during pregnancy but did not have orders dated during their third trimester (i.e. no third trimester exposure) also were associated with lower rates of adaptation syndrome compared to those who had escitalopram exposure occurring throughout pregnancy. Thus, for patients prescribed those two drugs, if depression symptoms tolerate stopping the drug or changing drugs in the third trimester, there may be a reduced odds of adaptation syndrome or NICU admission. For patients taking bupropion or escitalopram early in pregnancy, a discussion with their provider about changing medications to one of the other ones studied may be reasonable. Overall, however, there were no clear differences in the main outcomes for women who lacked exposure compared to those with exposure throughout pregnancy with the other drugs studied. If confirmed, these findings may provide some reassurance to patients and providers that they may not need to make a potentially difficult choice between adequate depression treatment during the third trimester and potential adverse effects for the newborn. Having no exposure in early, late, or both periods did not have significant associations with better or worse rates of outcomes for most drugs.

Adaptation syndrome is generally considered to be diagnosed in 9–28% of infants exposed to SSRI/SNRI antidepressants [10–12]. Our observed rates generally fell in this range. Overall, we did not find that lack of prescription-based exposure for any of the individual antidepressants in the third trimester reduced the risk of adaptation syndrome, supporting some previous work [11, 13]. For women taking escitalopram, however, we observed an association with a reduced odds of both adaptation syndrome and NICU admission when there were no third trimester prescriptions. This may warrant discussions with pregnant patients who have depression symptoms controlled on that drug. A similar example in support of this finding can be seen in a report for fluoxetine which demonstrated a higher rate of adaptation syndrome with late exposure compared to early exposure [10]. Our study results may have differed for fluoxetine, however, in that we compared women without a prescription in the third trimester to women prescribed the drug throughout pregnancy. A recent meta-analysis reported overall higher odds of specialized care admission with SSRI use (OR 1.74, 22% admission rate for exposed infants vs. 11% for unexposed) [14]. As all women in our cohort were exposed at some point during pregnancy, our rates are similar to the 22% reported in the meta-analysis.

Depression is associated with multiple pregnancy complications and is theorized to have an impact on newborn development as well [15]. Different drugs work better or worse for different individuals. Once drug therapy is indicated for a pregnant woman or any woman trying to conceive, it is paramount that providers have evidence-based risk and benefit information to discuss with the patient. These data may help give some reassurance that if a pregnant woman is deemed appropriate to initiate one of these SSRI/SNRI drugs, it may not be necessary to delay starting treatment until the first trimester is over nor to stop the drug once the third trimester arrives, potentially precipitating worsened depression, not to mention the potential impact on risk of developing postpartum depression. Our findings suggest that escitalopram may be an exception to this. Preparing a pregnant woman for the possibility of NICU admission or diagnosis of adaptation syndrome is part of antenatal counseling and can be done in a multidisciplinary fashion [6]. This way, informed, adequate depression treatment and antenatal care can happen hand-in-hand.

Our findings should be replicated in other large cohorts of women prescribed antidepressant drugs. Additionally, the ability to ensure adherence by women prescribed these medications would be important for other studies. This could be paired with drug concentrations in both mother and newborn. It has been demonstrated that adaptation syndrome is associated with antidepressant drug levels in umbilical cord blood [16]. Prospective evaluation of multiple antidepressants in a cohort of pregnant women with depression with detailed covariate information will be important to further enhance evidence-based counseling.

Access to an electronic database reflecting current clinical practice in a large health system is a strength which allowed for analysis of some less common neonatal outcomes and the ability to compare effects for individual antidepressants prescribed at different times during the pregnancy. Additionally, the ability to compare exposures occurring at multiple different timepoints in pregnancy using extracted data can help inform clinical counseling.

Because prescription records pulled from pharmacy datasets were used to represent timing of exposure in a subject, it is possible that subjects had not filled and/or taken the medication(s) of interest, resulting in a potential overrepresentation of exposure for any of the drugs in this study. It is well known that patients often do not take prescribed medication. Similarly, the pregnant woman’s gestational age at the time a medication was ordered could result in a prescription(s) lasting throughout an entire exposure period in this study (pre-pregnancy, first, second, and third trimesters) and not required a new order in the subsequent exposure period, potentially creating underrepresentation or mis-categorization of exposure. We attempted to take refills into account in our categorization. Lack of dosage information prevented the consideration of prescription strength as a possible factor in this study. We were unable to capture prescriptions outside of the systems queried by the Regenstrief Institute and we were unable to account for other drugs taken at baseline, such as opioids or benzodiazepines. These drugs, and others, could be potential confounders of the relationships. It is possible other confounders were unaccounted for as well. These may be addressed in future work. Potential coding errors encountered by utilizing data from large record-based data sets may also be a limitation, although the methodology employed by the Regenstrief Institute has been refined and validated over several decades [9]. We could not account for potential differences in patients who may be willing to stop medications later in pregnancy and those who continue to have prescriptions throughout pregnancy. A small proportion of obtained data was verified and found to be consistent with manual review of the electronic medical record. However, we did not review all clinical notes to verify if clinicians noted that the patient was taking the medication. Under-capture of data due to the methodology of using ICD9/10 codes cannot be ruled out, though rates of outcomes obtained (preterm birth (15.2%) and NICU admission (26.4%)) aligned with other published reports of antidepressant exposure in pregnant women [17]. Our rates of any adaptation syndrome (5–10%), however, were lower than other reports [10, 12, 18]. The change in diagnoses associated with SSRIs for NAS and PAS necessitated that both diagnosis be combined as ‘any adaptation syndrome’ for the purposes of this study. Thus, reported rates of adaptation syndrome in this study could be artificially high due to potential NAS associated with opioid or other antidepressant use. Under-capture due to reliance on administrative data is more of a possibility. While some newborns diagnosed with adaptation syndrome are admitted to the NICU, studies show that this rate can be less than 5% [19]. The relatively small proportion of Hispanic women (relative to the general population) limits the generalizability of our results. There is also potential for bias due to conditioning on future exposure. The power of this study for individual drugs and timing of exposure does not accommodate analysis of rarer adverse outcomes, such as congenital anomalies.

## Conclusion

For the majority of SSRI/SNRI drugs used to treat depression in pregnancy, exposure during different time points during pregnancy (compared to continued exposure throughout pregnancy) was not associated with change in rate of newborn outcomes of NICU admission or adaptation syndrome diagnosis. The only exceptions were potentially lower rates of NICU admission for both bupropion and escitalopram and lower rates of adaptation syndrome for escitalopram when no prescriptions were ordered in the third trimester (i.e. no third trimester exposure to either bupropion or escitalopram, in this case). These association data may help pregnant women with depression and in need of antidepressant therapy to have informed discussions with their providers about the potential risks and benefits of continuing or stopping drugs at different times during pregnancy.

## Supplementary Information

**Additional file 1: Appendix Table S1.** ICD9 and ICD10 codes and sources used to extract data. **Appendix Table S2.** Rates of additional newborn outcomes by drug exposure.

## Data Availability

The dataset supporting the conclusions of this article, including individual participant data will be made available to investigators upon request. All data are deidentified. All characteristics listed in the methods are able to be shared. No other documents are available. Data will be available 3 months after publication of the manuscript. Access requests should be made to the Corresponding author, Dr. Haas, at dahaas@iu.edu. Access requests will be discussed among the authors and the Regenstrief Institute. Any access requests must be accompanied by IRB approval for any proposed analyses. Data have not been deposited into a public repository due to local restrictions.
