# Supplementary material for: Does lack of exposure to individual antidepressants at different points during pregnancy associate with reduced risk of adverse newborn outcomes?
Source: BMC Pregnancy Childbirth. 2022 Dec 9;22:926. doi: 10.1186/s12884-022-05287-6 (PMC9733325; doi:10.1186/s12884-022-05287-6)
Supplement: Supplementary file 1 — Additional file 1: Appendix Table S1. ICD9 and ICD10 codes and sources used to extract data. Appendix Table S2. Rates of additional newborn outcomes by drug exposure. [file 12884_2022_5287_MOESM1_ESM.docx]

**Appendix Table S1.** ICD9 and ICD10 codes and sources used to extract data.

| **Variables** | **Units** | **ICD9** | **ICD10** | **Notes on search parameters** |
| --- | --- | --- | --- | --- |
| **Maternal** | | | | |
| **Age** | Years |  |  | At time of infant delivery |
| **Race/ethnicity** | Categorical |  |  | Categorized in record as Asian, Black, Hispanic, White, other, mixed |
| **Insurance** | Categorical |  |  | Commercial, Government (Medicare, Medicaid, Tricare), or None/Self-pay |
| **Due date (EDC)** | Date |  |  | The EDC assigned for the pregnancy |
| **History of prior preterm birth** | Yes/No | V13.21 | Z87.51 |  |
| **Drug prescribed and date** |  |  |  | Name of SSRI/SNRI drug and date prescribed |
| **Any other drug(s) ordered during the pregnancy** |  |  |  | List other drugs and order dates- limited to during calculated time of pregnancy |
| **History of Diabetes Mellitus** | Yes/No | 250.XX | E10.XXX--E13.XXX | To include Type 1, Type 2, other |
| **GDM** | Yes/No | 648.0X | O24.XXX |  |
| **History of Hypertension** | Yes/No |  | I10.XXX |  |
| **HDP** | Yes/No | 642.XX | O10.XX, O11.X, O13.X, O14.XX, O15.XX, O16.X |  |
| **Newborn outcomes** | | | | |
| **Gestational age at birth** | Weeks |  |  | Calculated using date of birth and known due date |
| **Date of birth** | Date |  |  | Delivery record |
| **Birth weight** | Grams |  |  | Delivery record |
| **Birth length** | Cm |  |  | Delivery record |
| **Birth head circumference (OFC)** | Cm |  |  | Delivery record |
| **Stillbirth** | Yes/No | V27.1 | P95 |  |
| **Dx of Postnatal Adaptation Syndrome** | Yes/No | 779.5 | P96.2XX or P04.15XX |  |
| **NICU admit** | Yes/No |  |  | Delivery record |
| **5-minute Apgar Score** | Numerical |  |  | Delivery record |
| **Jaundice needing treatment** | Yes/No | 774.6 | P58.9, P59.9 |  |
| **Dx of TTN of the newborn or RDS** | Yes/No | 769, 770.6 | P22.X |  |
| **Oxygen support (hypoxemia of newborn)** | Yes/no | 770.88 | P84 |  |
| **Date of discharge** | Date |  |  | Discharge summary |
| **Persistent PPH** | Yes/No | 747.83 | P29.30 |  |
| **Neonatal seizures** | Yes/No | 779.0 | P90 |  |
| **Cardiac malformations** | Yes/No | 745.XX, 746.XX | Q20.X, Q21.X, Q22.X, Q23.X, Q24.X, Q25.X, Q26.X, Q27.X, Q28.X |  |

X in the table are placeholders for any digits which would satisfy the diagnostic coding. When there are three Xs, it could be that there is one digit, two digits, or three digits. Drugs to capture: SSRIs (Citalopram, Escitalopram, Fluoxetine, Paroxetine, Sertraline) and SNRIs (Bupropion, Desvenlafaxine, Duloxetine, Venlafaxine).

GDM = gestational diabetes, HDP = hypertensive disorder of pregnancy, Dx = diagnosis, TTN = transient tachypnea of the newborn, RDS = respiratory distress syndrome, PPH = pulmonary hypertension of newborn

**Appendix Table S2.** Rates of additional newborn outcomes by drug exposure.

| **Outcome** | **Bupropion**  n=315 | **Citalopram** n=308 | **Escitalopram** n=470 | **Fluoxetine** n=470 | **Sertraline** n=1443 | **Overall cohort** N=3006 |
| --- | --- | --- | --- | --- | --- | --- |
| **Mean Birth weight (g)** | 3215.5 (947.0) | 3225.3 (589.7) | 3234.6 (577.1) | 3169.1 (600.7) | 3238.8 (595.1) | 3223.3 (595.4) |
| **Mean Birth length (cm)** | 49.8 (3.1) | 49.9 (4.6) | 50.0 (4.4) | 49.8 (3.8) | 50.0 (3.9) | 49.9 (4.1) |
| **Mean Birth Head circumference (cm)** | 34.2 (2.5) | 33.8 (2.5) | 34.1 (2.2) | 33.8 (2.6) | 34.0 (2.2) | 34.0 (2.3) |
| **5-minute Apgar score (median)** | 9 | 8 | 8 | 8 | 9 | 8 |
| **Jaundice requiring tx** | 51 (23.7%) | 43 (14.0%) | 78 (16.6%) | 73 (15.5%) | 207 (15.5%) | 452 (15.0) |
| **PPHTN** | 0 (0%) | 1 (3.2%) | 2 (0.4%) | 2 (0.4%) | 2 (0.1%) | 7 (0.2%) |
| **Neonatal seizures** | 1 (0.3%) | 1 (0.3%) | 3 (0.6%) | 3 (0.6%) | 4 (0.3%) | 12 (0.4%) |
| **Cardiac malformations** | 10 (3.2%) | 15 (4.9%) | 20 (4.2%) | 22 (4.7%) | 57 (4.0%) | 124 (4.1%) |

Data are presented as n (%) for discreet variables and as mean (standard deviation) for continuous variables.

Tx = treatment, PPHTN = Persistent pulmonary hypertension of the newborn
